# UHPLC-HR-MS/MS-Guided Recovery of Bioactive Flavonol Compounds from Greco di Tufo Vine Leaves

**DOI:** 10.3390/molecules24193630

**Published:** 2019-10-08

**Authors:** Simona Piccolella, Giuseppina Crescente, Maria Grazia Volpe, Marina Paolucci, Severina Pacifico

**Affiliations:** 1 Department of Environmental Biological and Pharmaceutical Sciences and Technologies, University of Campania “Luigi Vanvitelli”, Via Vivaldi 43, 81100 Caserta, Italy; simona.piccolella@unicampania.it (S.P.); giuseppina.crescente@unicampania.it (G.C.); 2 Istituto di Scienze dell’Alimentazione, Consiglio Nazionale delle Ricerche (CNR), 83100 Avellino, Italy; mgvolpe@isa.cnr.it (M.G.V.); paolucci@unisannio.it (M.P.); 3 Dipartimento di Scienze e Tecnologie, via De Sanctis, snc, 82100 Benevento, Italy

**Keywords:** food waste recovery, grape leaves, UHPLC-HR-MS/MS analysis, flavonol glycuronides recovery

## Abstract

Leaves of *Vitis vinifera* cv. Greco di Tufo, a precious waste made in the Campania Region (Italy), after vintage harvest, underwent reduction, lyophilization, and ultrasound-assisted maceration in ethanol. The alcoholic extract, as evidenced by a preliminary UHPLC-HR-MS analysis, showed a high metabolic complexity. Thus, the extract was fractionated, obtaining, among others, a fraction enriched in flavonol glycosides and glycuronides. Myricetin, quercetin, kaempferol, and isorhamnetin derivatives were tentatively identified based on their relative retention time and TOF-MS^2^ data. As the localization of saccharidic moiety in glycuronide compounds proved to be difficult due to the lack of well-established fragmentation pattern and/or the absence of characteristic key fragments, to obtain useful MS information and to eliminate matrix effect redundancies, the isolation of the most abundant extract’s compound was achieved. HR-MS/MS spectra of the compound, quercetin-3-*O*-glucuronide, allowed us to thoroughly rationalize its fragmentation pattern, and to unravel the main differences between MS/MS behavior of flavonol glycosides and glycuronides. Furthermore, cytotoxicity assessment on the (poly)phenol rich fraction and the pure isolated compound was carried out using central nervous system cell lines. The chemoprotective effect of both the (poly)phenol fraction and quercetin-3-*O*-glucuronide was evaluated.

## 1. Introduction

Food by-products and waste exploitation practices are gaining a lot of attention as these materials are an untapped but rich source for the recovery of bioactive compounds, favorably relevant for other food and feed scopes [1,2,3,4]. In fact, a consistent and recent literature highlights that valorizing agrofood wastes is not only a considerable alternative to composting, but also, and above all, a highly sustainable opportunity for obtaining added-value molecules, which could be efficaciously exploited in the nutraceutical and/or cosmeceutical sector, through an integrated approach involving multiple actors for an ecofriendly industrial development [5]. Phenols, carotenoids, and some other beneficial phytochemicals, together with pectin, are just a few examples of bioactives in agrofood wastes [6]. In particular, phenols and polyphenols, commonly found in high amounts in fruit and vegetable waste products, are broadly hypothesized to be used as natural food and drink preservatives, thanks to their ability to extend the expiration date of a product, thus delaying its rancidity and/or avoiding alteration of taste or other organoleptic characteristics [7]. Moreover, the pectin advantageous recovery makes the molecule largely exploitable as a gelling agent in pastry or as a fat replacement in meat products, or a binder in animal feed [8,9].

Considering that grape cultivation is one of the main agro-economic activities worldwide, with over 60 million tons produced globally every year [10], and that the entire wine production chain includes not only the production of grapes, their processing, and marketing, but also a large amount of wastes, it is reasonable to hypothesize fruitful recycle processes that go far beyond those already involved in the transformation of a grape waste part (such as that destined for distilleries) [11]. In fact, although during the wine-making process, different by-products are generated and found to be valuable for alternative use in a new production cycle (e.g., stems, grape-marc, and grapeseed) [12], waste and effluents, normally rich in sugars, proteins, fibres, and lipids as well as vitamins and other bioactive compounds, are also generated. Thus, they could represent an ideal source for obtaining chemicals and pharmaceuticals with high added value, as well as for creating biomaterials and substrates that can be used in different biological processes [13].

However, at the national and international level, an integrated approach, that allows a complete recovery of oenological wastes through the development of efficient and sustainable technologies from an economic and environmental point of view, leading to the final production of different products with standard features and demonstrating applications in specific sectors, is still lacking. Although marc and grapeseed already have an acclaimed use, even the leaves of the vine can be considered as the stalk, a curious waste to be recovered in the vine and wine industry [14]. Leaves, which unlike dregs, marc (skins and grape seeds), and stalks, are not included in the Italian Ministerial Decree from 27 November 2008 as renewable by-products, are waste material whose disposal during fruit harvesting is massive, not only in the early stages of winemaking, but mainly in destemming. Pre-bloom leaf removal, which consists of removing all or part of the leaves from the fruitful area in the period from spring to late season, is a practice commonly used with the aim of improving the quality of the harvest. On the other hand, countries such as Florida, Greece, and many Middle Eastern countries use the cultivation of vines also for the production of leaves used in kitchens for the preparation of typical dishes (e.g., the Arabian warak enab dish) in the knowledge that, like fruits, they contain numerous substances beneficial to humans such as organic acids, vitamins, and stilbenes [15]. The vine leaves are also rich in anthocyanins and tannins with vasoactive and vasoprotective properties and which have the ability to stimulate vascularization [16].

In this context and with the aim to exploit the recovery of bioactive molecules from wine production wastes, leaves of *Vitis vinifera* cv. Greco di Tufo, collected in the Campania Region (Italy), were considered. The production of this wine with the denomination of controlled and guaranteed origin represents a great resource of the territory. This ancient vine, whose name derives from the characteristic shape of the bunches, with grapes grouped in pairs, was introduced by the Greeks along the Tyrrhenian coasts. The geographical environment favors the production of this prized wine with a characteristic flavor.

Leaves were extracted through maceration and the alcoholic extract obtained was further fractionated. The phytoextract chemical composition was unravelled by UHPLC-HR-MS/MS analysis. The powerful analytical tool was thoroughly investigated for achieving suitable and valid information on the spectral behavior of Greco di Tufo leaf metabolites. The extract, and the most abundant compound isolated therefrom, were further evaluated for their potential cytotoxicity in SH-SY5Y neuroblastoma and U-251 MG glioblastoma cell lines.

## 2. Results and Discussion

The valorization of Greco di Tufo vine leaves, an unexplored source of bioactive molecules, took advantage of the design of a green and sustainable extraction method, pursued using ethanol, the most common biosolvent. The fraction GrM was broadly analyzed for its bioactives using UHPLC-HR-MS/MS tools, and for its cytotoxic effects. The lack of toxic effects and its ability to inhibit acetylcholinesterase enzyme, together with the observation of its richness in glucuronidated flavonols, with dissimilar fragmentation pattern in respect to that of the most common glycosides, laid the foundation for the phytochemical investigation of the extract and the purification of the most abundant compound, quercetin-*O*-glucuronide (GrM_1_). The phytochemical approach represented a useful strategy to define the flavonol glucuronides’ MS/MS chemical behavior for the rapid identification of these compounds.

### 2.1. GrM Chemical Composition

**Figure 1:**
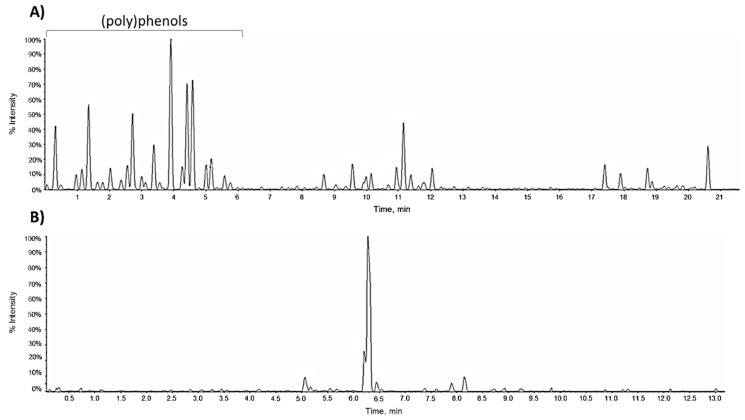
TIC (Total Ion Chromatogram) of (**A**) EtOH extract and (**B**) GrM fraction from Greco di Tufo vine leaves.

The alcoholic extract from the leaves of *Vitis vinifera* cv. Greco di Tufo, as evidenced by a preliminary UHPLC-HR-MS analysis, showed a high metabolic complexity. The extract was rich in (poly)phenol, alkylphenol, glycerolipid and glycerophospholipid components (Figure 1A).

The parental extract was further fractionated by normal-phase column chromatography, using three solvents with increasing polarity. Among the fractions obtained, the alcoholic one, named GrM, was peculiarly enriched in flavonol glycosides and glycuronides (Table 1; Figure 1B). Flavonol hexuronides, not massively produced in the plant environment, are commonly described as bioconversion products of the phytochemicals taken with the diet or introduced by supplementation with less toxicity [17]. Indeed, their presence is not negligible in plants with common analytical techniques. In fact, these compounds, whose chemical structure was deeply elucidated by NMR spectroscopy, were also isolated from *Vitis × Labruscana* cv. ‘Isabella’ leaf methanol crude extract [18]; recently, their presence was suggested as part of the minor components in hemp seed oil [19].

**Figure 2:**
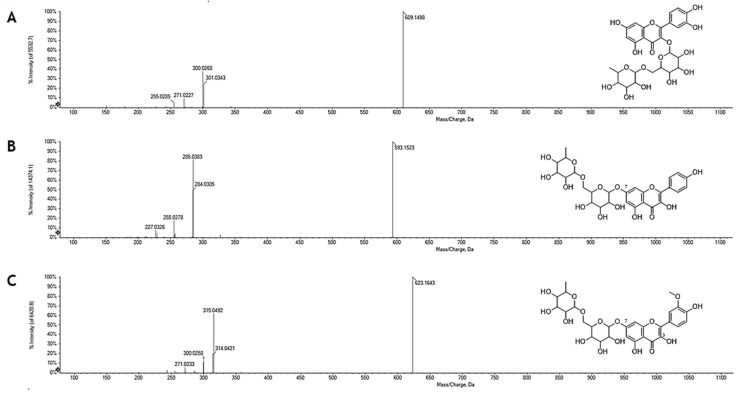
Flavonol rutinosyl derivatives; TOF-MS^2^ spectrum of metabolites (**A**) **12**; (**B**) **15**; and (**C**) **17.**

Based on the relative retention time and the TOF-MS^2^ data, five derivatives of myricetin (**4**–**7**, **9**), three derivatives of quercetin (**12**–**14**), two derivatives of kaempferol (**15**,**16**) and two derivatives of isorhamnetin (**17,18**) have been tentatively identified (Table 1). The neutral loss of 162.05, 176.03 and 308.11 Da was in accordance with hexosyl, hexuronidyl and disaccharidic derivatives of the four flavonols. In particular, the neutral loss of 308.11 Da allowed us to hypothesize, for the metabolites **12**, **15** and **17**, a deoxyhexose and hexose moiety, which on the basis of the relative intensity of the radical aglycone ion ([aglycone–H]^•–^) and [aglycone–H]^–^, was linked to the –OH function in C-3 of the flavonolic nucleus in **12**, and to the phenolic function in C-7 in **15** and **17** (Figure 2). The identity of the flavonol glycoside **12** as rutin (rutinosyl derivative of quercetin) was further estimated by comparing the retention time and fragmentation pattern with that of the reference pure compound.

**Figure 3:**
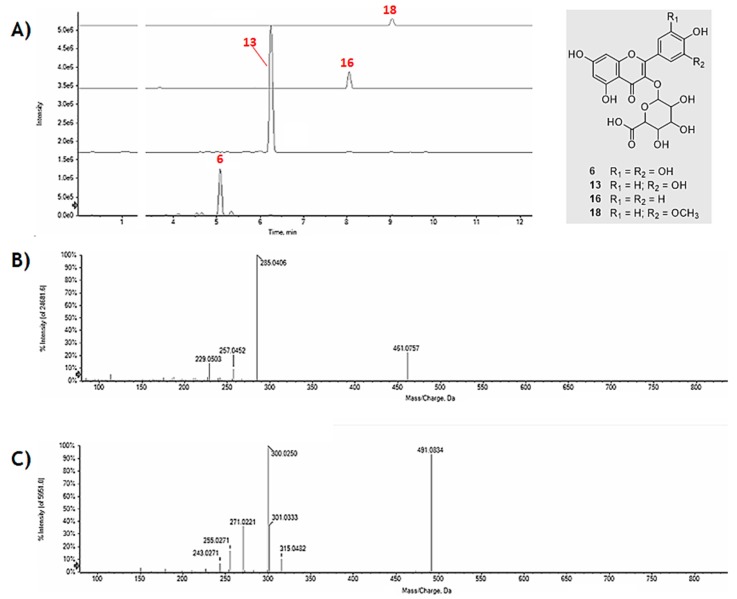
(**A**) Extracted ion chromatograms (XICs) of hexuronyl flavonols, whose structure is depicted in the grey box; TOF-MS^2^ spectra of (**B**) **16** and (**C**) **18**.

The neutral loss of 176.03 Da, corresponding to a dehydrated hexuronic acid, characterized the MS/MS spectra of metabolites **6**, **13**, and **16**, whose deprotonated molecular ion dissociated providing the product ion [aglycone–H]^–^ as base peak; the only exception was represented by compound **18**, for which the most favourable CH_3_^•^ loss gave an abundant ion at *m*/*z* 300.0250 (Figure 3).

**Figure 4:**
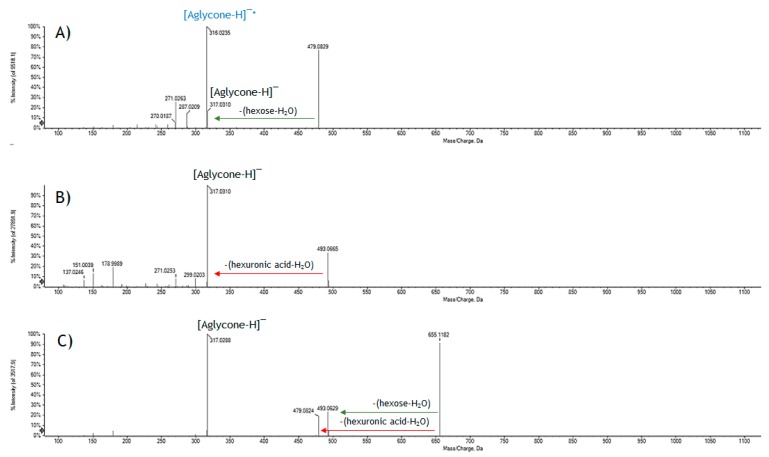
TOF-MS^2^ spectra of metabolites (**A**) **5**; (**B**) **6**; and (**C**) **9**.

The lack of well-established fragmentation pattern and/or the absence of characteristic key fragments make difficult the localization of the hexuronyl moiety. In fact, the main fragment detected, for example, for the abundant metabolite **13** was that corresponding to the deprotonated aglycone quercetin, as well as other characteristic ions of the flavonol such as those at *m*/*z* 273.0399, due to CO loss ([aglycone-28]^–^), and at *m*/*z* 255.0293, which showed a relative intensity of only 5.1% and 8.5%, respectively. Other characteristic fragments of quercetin were identified in the ions at *m*/*z* 151.0029 and 178.9979 corresponding, respectively, to the deprotonated A ring, released by a retro-Diels Alder mechanism, and to the product of retrocyclization on bonds 1 and 2 [20]. A similar behavior was evident for the other hexuronyl derivatives and in particular, for those of myricetin. In Figure 4, the TOF-MS^2^ spectra of myricetin derivatives **5**, **6** and **9** are reported; they were tentatively identified as myricetin hexoside, hexuronide and hexosyl hexuronide, respectively. It is evident that the presence of an hexuronyl moiety massively influences the fragmentation of the aglycone, impoverishing in intensity its characteristic ions.

### 2.2. GrM_1_ Purification

**Figure 5:**
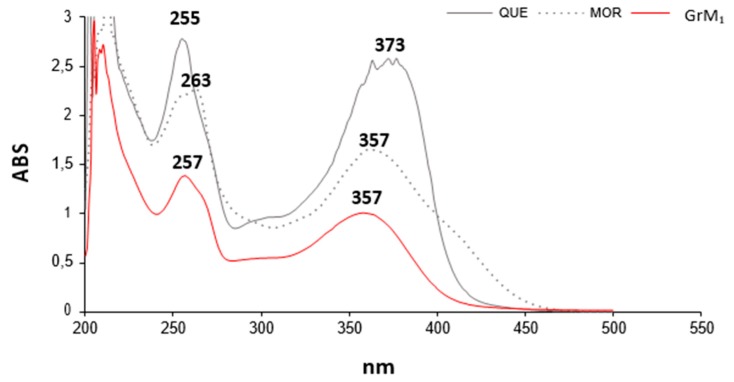
Comparison of the UV–Vis spectra of the metabolite GrM_1_ and of quercetin (QUE) and morin (MOR) flavonol isomers.

To obtain useful MS information and to eliminate matrix effect redundancies, a GrM fraction aliquot underwent thin-layer chromatography, which yielded, among others, the metabolite GrM_1_ (the compound **13** of the GrM mixture). The UV–Vis spectrum of the molecule confirmed the presence of a flavonol skeleton molecule (Figure 5). In fact, flavonols, like flavones, present two major absorption peaks (λ_max_) in the region between 240–280 nm (commonly referred to as band II) and between 300–380 nm (band I). This latter band favors the distinction of the two classes of flavonoids since the flavone λ_max_ is between 304–350 nm, while that of the flavonols is between 352–385 nm. Band I is associated with the absorption of the cinnamoyl system and band II with that of the benzoyl system (ring A). Literature evidence suggests that when glucuronidation occurs at the C-3 position, a hypsochromic shift of band I of about 14–29 nm is observed, whereas the glucuronidation at the phenolic function in C-7 does not lead to variations if not minimal or void [21]. GrM_1_ spectrum showed, compared to the standard quercetin, a blue shift of the band I of 16 nm and a red shift of band II of 2 nm according to the quercetin glucuronidate in C-3.

**Figure 6:**
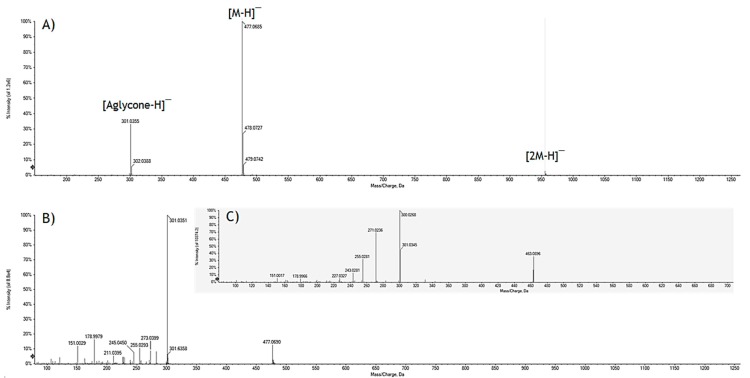
**(A**) TOF-MS and (**B**) TOF-MS^2^ spectra of the quercetin-3-*O*-glucuronide. (**C**) Quercetin-3-*O*-glucoside TOF-MS^2^ spectrum.

The TOF mass spectrum of the molecule is completely superimposable to that recorded for peak **13** of the mixture (Figure 6) with the deprotonated molecular ion at *m*/*z* 477.0685, the ion [2M − H]^–^ at *m*/*z* 955.1420 and, again, the ion [aglycone − H]^–^ at *m*/*z* 301.0355. The TOF-MS^2^ spectrum of the ion at *m*/*z* 477.0685 provided the ion [aglycone − H]^–^ as base peak and the ions [aglycone – H_2_O – H]^–^ (*m*/*z* 283.0244), [aglycone − CO − H]^–^ (*m*/*z* 273.0399), [aglycone – CO − H_2_O − H]^–^ (*m*/*z* 255.0293) and [aglycon e− 2CO − H]^–^ (*m*/*z* 245.0450), all with an intensity lower than 10%.

**Figure 7:**
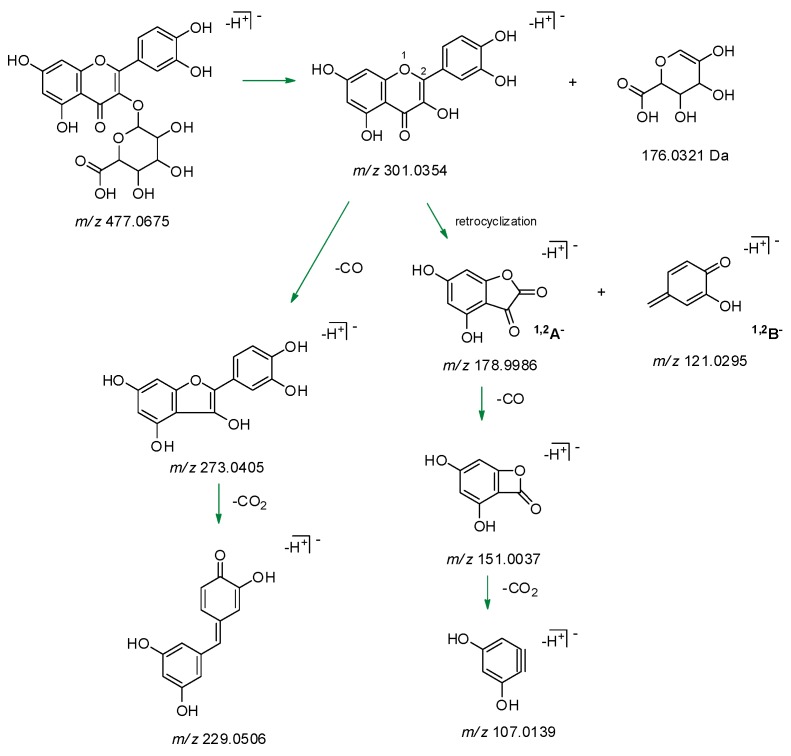
Proposed fragmentation pathway of GrM_1_ [aglycone – H]^–^ ion. Theoretical *m*/*z* values are reported.

When [aglycone – H]^–^ ion dissociated (spectrum not shown), it provided, with relative abundance of 20%, the ion at *m*/*z* 151.0039 (calcd. 151.0337). The latter is the result of a loss of CO from the ion at *m*/*z* 178.9988 (calcd. 178.9986), whose presence, together with that of the ion at *m*/*z* 121.0300 (calcd. 121.0295), confirmed the presence of the flavonol quercetin. In fact, the two ions were attributable to the retrocyclization that is realized between the bonds 1 and 2 of the flavonol nucleus with formation of the fragments ^1,2^A^–^ and ^1,2^B^–^ (Figure 7). The loss of a CO_2_ unit from the ion at *m*/*z* 151.0039 provided the ion at *m*/*z* 107.0134 (calcd. 107.0139). Comparing the spectrum of the standard quercetin with that obtained by GrM_1_ deprotonated aglycone dissociation, the two realities were fully superimposable.

**Figure 8:**
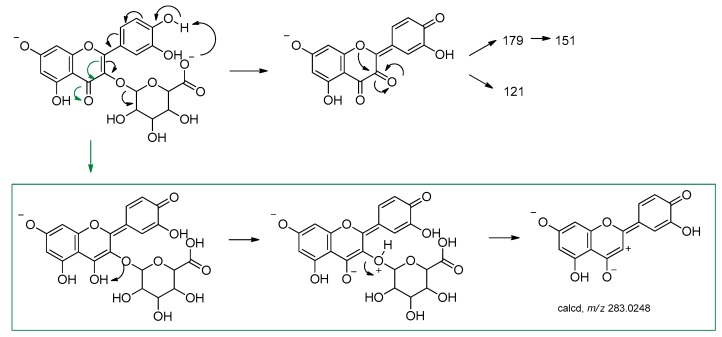
Proposed mechanism for the formation of the ion at *m*/*z* 283.0249. The calculated *m*/*z* value is reported (error < 5 ppm).

The [aglycone−H_2_O−H]^–^ ion (*m*/*z* 283.0244), detected in GrM_1_ TOF-MS^2^ spectrum, could also represent a characteristic fragment defining the localization of the hexuronic acid in C-3 of the aglycone (Figure 8). The electronic delocalization on oxygen in C-4 could favor the formation of an enolic function, the proton abstraction with the formation of a good leaving group, whose detachment defines the formation of an anion in which there is a charge separation.

### 2.3. Cytotoxicity of GrM

Glucuronidated flavonoids display important health properties [22,23]. Baicalein 7-*O*-β-glucuronide was observed to promote wound healing and to exert antitumor activity [24,25]; quercetin 3-*O*-β-glucuronide is anti-inflammatory and neuroprotective, whereas 3-methoxyflavonol-4′-*O*-glucuronide is anti-allergenic and epicatechin glucuronide promotes vascular function. Glucuronidation greatly affects flavonoids’ physiological properties, frequently their solubility and thus bioavailability. Bioactivity is differently influenced by glucuronidation and glucuronate moiety localization as it could be increased or decreased, whereas intra- and extra-cellular transport, understood as excretion, commonly increases [16]. In particular, it was reported that the oral administration of a blend of vine supplements is effective in protecting against neuropathologies and cognitive impairment that occurs with aging. Based on this evidence, the cytotoxicity of the GrM extract on the SH-SY5Y cell line was preliminarly evaluated. The choice of this cell line is based on its common use in in-vitro studies related to neurotoxicity and neurodegenerative diseases. Indeed, it is evident that primary cultures would be the best choice for this kind of investigation, as they are able to mimic the properties of neuronal cells in vivo. However, the preparation and culture of primary cells is much more challenging, especially for neuronal cells [26].

**Figure 9:**
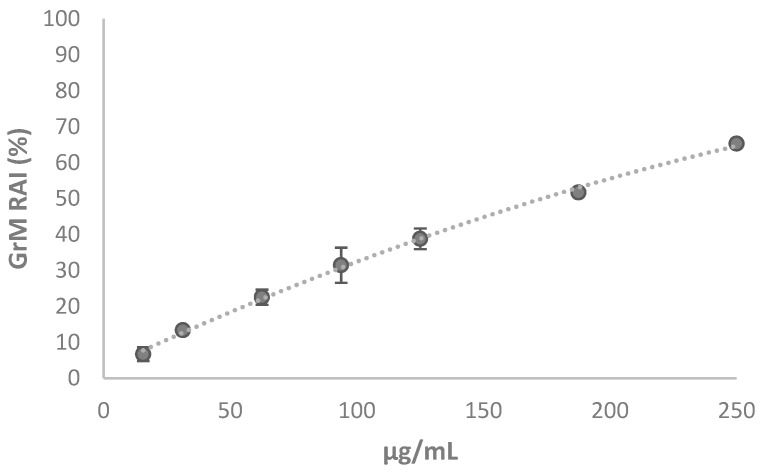
Redox mitochondrial inhibition (RAI%) of GrM extract towards SH-SY5Y cell line at 48 h exposure time. Values are reported as mean ± SD of measurements carried out on 3 samples (n = 3) analyzed 12 times.

The 3-(4,5-dimethyl-2-thiazolyl)-2,5-diphenyl-2H-tetrazolium bromide (MTT) test was used for this purpose. It is able to measure the capacity of the mitochondrial dehydrogenases to reduce the tetrazolium ring of MTT, yellow colored, generating a chromogenic compound, the purple formazan. Obviously, this conversion is possible only in metabolically active cells. The results of the MTT assay suggested that the extract at doses ranging from 15.6 to 62.5 μg/mL did not massively influence the activity of mitochondrial dehydrogenases and a weak inhibitory effect on mitochondrial redox activity, equal to 31% and 38%, is recorded at the doses of 93.75 and 125 μg/mL, respectively (Figure 9).

The inhibition resulted was dose and time dependent and reached 62.1% of redox activity inhibition (RAI) for exposure to the highest tested dose. The lack or weak toxicity of the extract, especially at low doses, together with the chemical constitution that sees the co-presence of notoriously antioxidant molecules, led us to undertake studies evaluating the inhibitory properties of acetylcholinesterase. Acetylcholinesterase (AChE) plays an important biological role in the termination of the nerve impulse at the level of cholinergic synapses by rapid hydrolysis of its substrate, acetylcholine. Our data, although preliminary, show that the phytocomplex at 25 μg/mL inhibits the activity of the enzyme (16.8 ± 3.2%) similar to donepezil (DP; 21.7 ± 1.8), one of the drugs most commonly used to increase memory function in patients with Alzheimer’s disease. The drug was tested, based on literature data, at the concentration of 3 μM, and was shown to exert an inhibition of cell proliferation of about 30% in SH-SY5Y cells [27].

### 2.4. Cytotoxicity of GrM_1_

**Figure 10:**
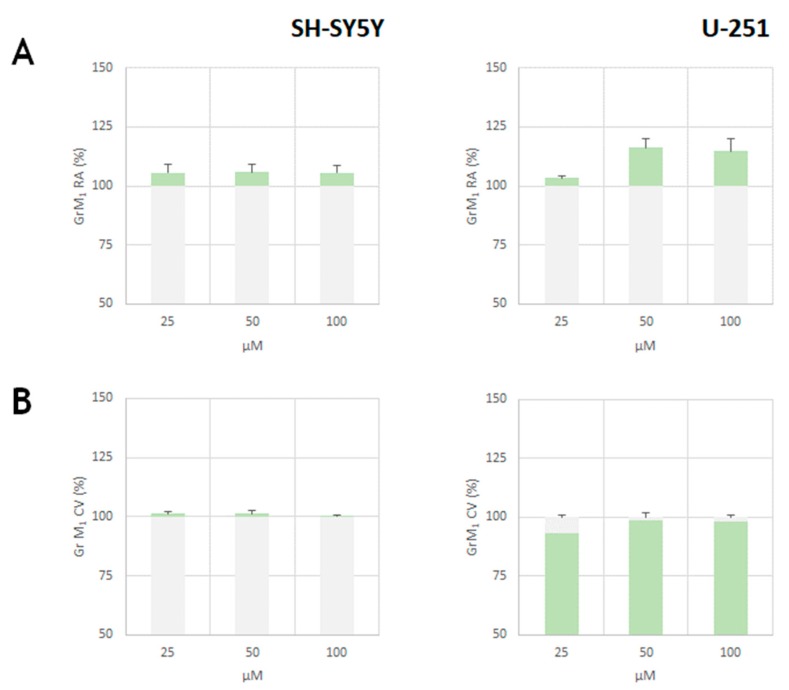
GrM_1_ cytotoxicity on SH-SY5Y cells and U-251 MG after 48 h exposure time. Mitochondrial redox activity (RA, %) was from MTT test data (**A**), whereas cell viability (CV, %) was from sulforhodamine B (SRB) test (**B**). Values are reported as mean ± SD of measurements carried out on 3 samples (n = 3) analyzed 12 times.

In order to verify the effects of the pure metabolite GrM_1_, SH-SY5Y cells were exposed to the molecule and their cell viability was evaluated by the MTT test (Figure 10A). The molecule, tested at 25, 50 and 100 μM doses, did not exert toxic effects on the activity of mitochondrial dehydrogenases. These cytoprotective effects were further confirmed in U-251 MG, one of the immortalized glial cell lines which could be used instead of primary culture systems as a model for neural cells, based on the assumption that they are more homogenous, thus providing more useful tools. Indeed, glial cells are known as supportive elements of the nervous system, providing an optimal microenvironment for neurons [28].

**Figure 11:**
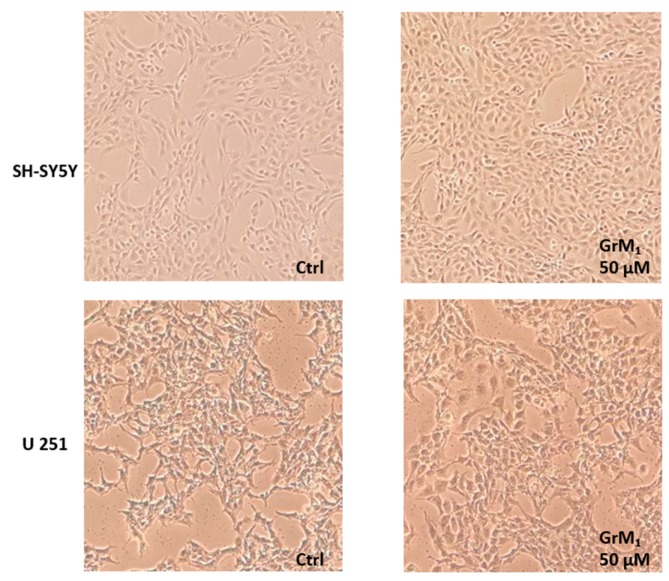
Morphological analysis of cells treated with GrM_1_ pure compound after 48 h exposure time.

The SRB (sulforhodamine B) test confirmed the absence of cytotoxicity (Figure 10B) and the weak proliferative effect was also verified by microscopic morphological change analysis (Figure 11).

**Figure 12:**
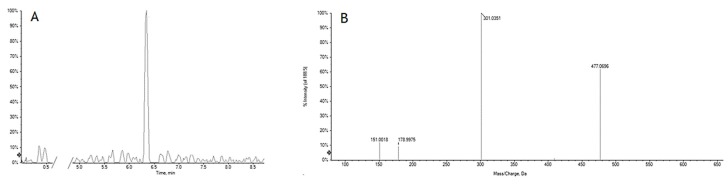
XIC (Extracted ion chromatogram) and TOF-MS^2^ spectrum of the ion at *m*/*z* 477.0696 from cell pellet extract.

Furthermore, after treating SH-SY5Y cells with GrM_1_ at 50 µM concentration, in a preliminary cell metabolomics scenario, the extraction of the cell pellet with a solution of MeCN:H_2_O (1:1, *v*:*v*), after appropriate quenching and extraction operation, highlighted a peak at *m*/*z* 477.0696, whose TOF-MS^2^ spectrum was super-imposable to that of GrM_1_ (Figure 12).

The data obtained were in agreement with the results previously reported in literature, according to which the bioconversion of quercetin and rutin in the glucuronidate derivatives is accompanied, in the HL-60 leukemic cells [29], by a complete elimination of the toxic effect commonly ascribed to the most common flavonols and preservation of their structural entity in the intracellular environment.

## 3. Materials and Methods

### 3.1. Plant Extraction and Fractionation

Leaves of *Vitis vinifera* cv. Greco di Tufo were collected in Montefusco (Avellino, Italy) in October 2017; the leaves were freeze-dried for 3 days using the FTS-System Flex-dry™ instrument (SP Scientific, Stone Ridge, NY, USA). Cryo-dried leaves were pulverized, using a rotary knife homogenizer and a sample (386.8 g) underwent solid-liquid extraction by maceration using ethanol as extracting solvent. Three extraction cycles (24 h each) were performed at 4 °C in the complete absence of light in order to obtain complete recovery of the metabolic content from *Vitis vinifera* cv. Greco di Tufo leaves. At the end of each cycle, the sample was filtered and the extraction solvent was removed using a rotary evaporator (Heidolph Hei-VAP Advantage, Schwabach, Germany). Ethanol extract was further fractionated by flash column chromatography (FCC) on Merck Kieselgel 60 (40–63 μm) silica gel under pure N_2_ pressure (h = 20 cm, Ø = 4.0 cm), eluting first with CHCl_3_, then with a CHCl_3_:EtOAc solution (1:1, *v*/*v*), subsequently with pure EtOAc, and finally with pure MeOH. The alcoholic fraction (20.63 g) was further chromatographed on Amberlite XAD-4 (h = 70 cm, Ø = 4.0 cm) eluting with water first (GrW) and then with MeOH (GrM). GrM fraction was analyzed by UHPLC-HR-MS.

**Figure 13:**
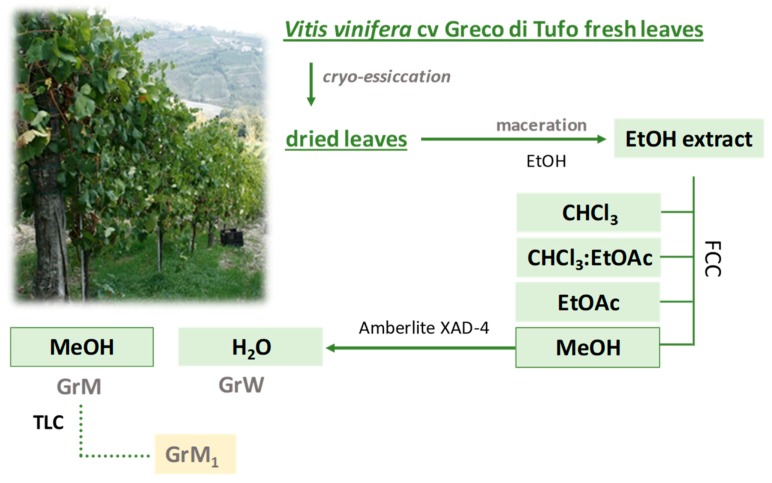
Extraction and fractionation scheme (FCC = flash column chromatography; TLC = thin-layer chromatography).

An aliquot of GrM fraction (36 mg) was chromatographed by thin-layer chromatography (TLC) using a precoated silica gel 60 F254 (20 × 20 cm, 1 mm, Merck, Darmstadt, Germany) and eluting with EtOAc:MeOH:H_2_O:HCOOH (16:2:1:1) solution. GrM_1_ (14.6 mg) was thus obtained and analyzed by UV–Vis and UHPLC-HR-MS. The fractionation scheme is depicted in Figure 13.

### 3.2. UHPLC-HR-MS and UV–Vis Analyses

GrM and GrM_1_ fractions, placed in vials at a concentration of 10 mg/mL in pure methanol UHPLC grade, were analysed by the Shimadzu NEXERA UHPLC system and the Omega Luna C18 column (50 × 2.1 mm, 1.6 μm). The mobile phase consisted of a binary solution A: 0.1% formic acid in water and B: 0.1% formic acid in acetonitrile. A linear gradient was used for the analysis: 0–5 min, 5 → 15% B; 5–10 min, 15% B; 10–12 min, 15 → 17.5% B; 12–15 min, 17.5 → 45% B; 15–16.50 min, 45% B; 16.50–16.51 min, 45 → 5% B; 16.51–18.00 min, 5% B. The injection volume was 2.0 μL and the flow was set at 0.5 mL/min. MS analysis was performed using the AB SCIEX TripleTOF 4600 (AB Sciex, Concord, ON, Canada) system with a DuoSpray ion source operating in negative electrospray ionization. The APCI (Atmospheric Pressure Chemical Ionization) probe of the source was used for fully automatic mass calibration using the calibrant delivery system (CDS). CDS injects a calibration solution matching polarity of ionization and calibrates the mass axis of the TripleTOF^®^ system in all the scan functions used (MS or MS/MS). Data were collected by information-dependent acquisition (IDA) using a TOF-MS survey scan of 100–1500 Da (250 ms accumulation time) and eight dependent TOF-MS/MS scans of 80–1300 Da (100 ms accumulation time), using a collision energy (CE) of 35 V with a collision energy spread (CES) of 25 V. The following parameter settings were also used: declustering potential (DP), 70 V; ion-spray voltage, –4500 V; ion source heater, 600 °C; curtain gas, 35 psi; ion source gas, 60 psi. Data processing was performed using the PeakView^®^-Analyst^®^ TF 1.7 Software.

UV–Vis spectrum of GrM_1,_ as well as those of the pure reference compounds quercetin and morin, were acquired in the range 200–600 nm by a Shimadzu UV-1700 double beam spectrophotometer (Kyoto, Japan).

### 3.3. Cell Culture and Cytotoxicity Assessment

Tests assessing cell viability and mitochondrial activity were performed to monitor the cytotoxic potential of the GrM fraction from *Vitis vinifera* cv. Greco di Tufo and of the purified GrM_1_. For this purpose, a stock solution of the two samples was prepared. Recorded activities were compared to an untreated blank arranged in parallel to the samples. Results are the mean ± SD values.

Human neuroblastoma cell line SH-SY5Y and glioma cell line U-251 MG were cultured in DMEM (Dulbecco’s Modified Eagle Medium) medium supplemented with 10% fetal bovine serum, 50.0 U/mL of penicillin and 100.0 μg/mL of streptomycin at 37 °C in a humidified atmosphere containing 5% CO_2_.

#### 3.3.1. MTT (3-(4, 5-Dimethylthiazolyl-2)-2,5-Diphenyltetrazolium Bromide) Cell Viability Test

Cells were seeded in 96-multiwell plates at a density of 1.0 × 10^5^ cells/well. After 24 h, cells were treated with different doses of the GrM extract (15.625, 31.25, 62.5, 93.75, 125.0, 187.5 and 250.0 µg/mL) and pure GrM_1_ metabolite (25, 50 and 100 µM) in a culture medium for 48 h. At the end of incubation, MTT (150 µL; 0.50 mg/mL in culture medium) was added. After 1 h at 37 °C in a 5% CO_2_ humidified atmosphere, MTT solution was removed and formazan was dissolved in dimethyl sulfoxide (DMSO) (100 µL). The absorbance at 570 nm was determined using a Tecan Spectra Fluor fluorescence and absorbance reader. Mitochondrial redox activity inhibition (RAI, %) was calculated using the following formula: [(Abs_untreated cells_)−(Abs_treated cells_)/(Abs_untreated cells_)] × 100, where Abs stands for absorbance [26].

#### 3.3.2. SRB (Sulforhodamine B) Cell Viability Test

Cells were seeded in 96-multiwell plates at a density of 1.0 × 10^5^ cells/well. After 24 h, cells were treated with pure GrM_1_ metabolite (25, 50 and 100 µM) for 48 h. At the end of incubation, cells were fixed with ice-cold trichloroacetic acid (TCA) (10% *w*/*v*, 40 μL) for 1 h at 4 °C. The plates were washed five times in distilled water and allowed to dry. Then, sulforhodamine B (SBR; 50 μL, 0.4% *w*/*v* in 1% aqueous acetic acid) was added to each well and incubated at room temperature for 30 min. In order to remove unbound dye, the plates were quickly washed with 1% aqueous acetic acid and dried subsequently. The bound SRB was solubilized by adding 100 μL of 10 mM unbuffered Tris base (pH 10.5) to each well and shaking for 5 min on a shaker platform. Finally, the absorbance at 570 nm of each well was measured using a Tecan SpectraFluor fluorescence and absorbance reader. Cell viability inhibition (CVI, %) was determined using the following formula: [(Abs_untreated cells_)–(Abs_treated cells_)/(Abs_untreated cells_)]×100, where Abs stands for absorbance [26,30].

#### 3.3.3. Anti-Acetylcholinesterase (AChE) Activity Assay

SH-SY5Y cells were treated with GrM at a dose level equal to 25 μg/mL and then a colorimetric test was carried out using an acetylcholinesterase assay kit (Colorimetric) (Abcam, UK) in accordance with the manufacturer’s instructions. The reaction was followed spectrophotometrically by the increase in absorbance at 412 nm. Donepezil (3 μM) was used as a positive standard [31].

#### 3.3.4. Cell Metabolomic Analysis

The SH-SY5Y and U-251 MG cells (2.5 × 10^6^ cells in Petri dish) were treated with the metabolite GrM_1_ for 48 h. At the end of the exposure period, the cells were first quenched in NaCl 0.9% in order to stop the metabolic activities. The cells were scraped and collected in Eppendorf tubes, centrifuged and extracted using 1.0 mL of a cold MeCN:H_2_O (1:1, *v:v*) solution. The extract was dried and reconstituted for UHPLC-HR-MS analysis [32].

## 4. Conclusions

In the present work the possibility of recovering bioactive components from the leaves of *Vitis vinifera* cv. Greco di Tufo was investigated. The employed extractive and fractionation procedures favored the preparation of an extract enriched in flavonol hexuronides variously oxygenated at the B-ring level.

The UHPLC-HR-MS/MS characterization of these molecules showed that the analytical protocol can be used for their rapid identification. The absence of cytotoxicity of the GrM and GrM_1_ extracts opens up new investigations at the cellular level aimed at ascertaining their neuroprotective potential for the functional recovery of a precious waste made in the Campania Region (Italy).

## Figures and Tables

**Table 1:** Metabolites tentatively identified in the GrM extract.

	Rt (min)	Tentative Assignment	Formula	[M − H]^–^ Found (*m*/*z*)	[M − H]^–^ calc. (*m*/*z*)	Error (ppm)	RDB	MS/MS Fragment Ions (*m*/*z*)
**1**	0.308	Gallic acid hexoside	C_13_H_16_O_10_	331.0666	331.0671	–1.3	6	169.014; 125.0248
**2**	3.129	Benzyl *O*-[arabinofuranosyl-(1-6)-glucoside]	C_18_H_26_O_10_	401.1460 437.1232 [M + Cl]^–^	401.1453	1.7	6	269.1030; 193.0486; 101.0238; 85.0279
**3**	4.204	Lariciresinol hexoside	C_26_H_34_O_11_	521.2043	521.2028	2.8	10	359.1507; 344.1273; 329.1035; 313.1078; 299.0936; 255.0657; 255.0687; 241.0509
**4**	5.086	Myricetin derivative	C_23_H_16_O_14_	515.0456	515.0467	–2.2	16	339.0125; 317.0300; 316.0225; 271.0225; 178.9976; 151.0018
**5**	5.091	Myricetin hexoside 1	C_21_H_20_O_13_	479.0840	479.0831	1.8	12	479.0829; 317.0310; 316.0235; 287.0209; 271.0263; 270.0187; 259.0268; 214.0280
**6**	5.094	Myricetin hexuronide	C_21_H_18_O_14_	493.0630	493.0624	1.3	13	493.0665; 317.0310 (178.9980; 151.0042; 137.0243; 109.0291); 299.0203; 271.0253; 227.0347; 178.9989; 151.0039; 137.0246
**7**	5.224	Myricetin hexoside 2	C_21_H_20_O_13_	479.0841	479.0831	2.1	12	317.0310; 316.0228; 287.0202; 271.0253; 270.0185; 259.0248; 242.0219; 214.0291; 178.9968; 151.0043
**8**	5.323	Furan/piran linalool oxide pentosyl hexoside	C_21_H_36_O_11_	463.2198 509.2253 [M + FA]^–^	463.2185	2.8	4	463.0896; 331.1754; 161.0447; 101.0240; 85.0289
**9**	5.424	Myricetin hexosyl hexuronide	C_27_H_28_O_19_	655.1143	655.1152	–1.4	14	655.1182; 493.0629; 479.0824; 317.0288
**10**	5.580	Caffeic acid derivative	C_22_H_36_O_12_	491.2190	491.2134	5.9	5	329.1618; 227.1272; 101.0242
**11**	5.731	Hydroxygeraniol pentosyl hexoside	C_21_H_38_O_11_	465.2353 501.2113 [M + Cl]^–^	465.2341	2.5	3	251.0794; 191.0578; 149.0447; 131.0352; 101.0241; 89.0246
**12**	6.270	Rutin	C_27_H_30_O_16_	609.1486	609.1461	4.1	13	609.1498; 301.0343; 300.0268; 271.0227; 255.0285
**13**	6.288	Quercetin hexuronide	C_21_H_18_O_13_	477.0685 955.1420 [2M – H]^–^	477.0675	3.2	13	477.0690; 301.0351; 283.0244; 273.0399; 255.0293; 245.0450; 211.0395; 178.9979; 151.0029; 121.0297; 107.0136
**14**	6.494	Quercetin hexoside	C_21_H_20_O_12_	463.0896	463.0882	3	12	463.0896; 301.0345; 300.0268; 271.0236; 255.0281; 243.0281; 227.0327; 178.9926; 151.0017
**15**	7.998	Kaempferol rutinoside	C_27_H_30_O_15_	593.1534	593.1512	3.7	13	593.1523; 285.0383; 284.0305; 255.0278; 229.0497; 227.0326
**16**	8.187	Kaempferol hexuronide	C_21_H_18_O_12_	461.0738	461.0725	2.7	13	461.0757; 285.0406; 257.0452; 229.0503
**17**	8.722	Isorhamnetin rutinoside	C_28_H_32_O_16_	623.1620	623.1618	0.4	13	623.1643; 315.0492; 314.0421; 300.0258; 271.0233
**18**	9.305	Isorhamnetin hexuronide	C_22_H_20_O_13_	491.0845	491.0831	2.8	13	491.0834; 315.0482; 301.0333; 300.0250; 271.0221; 255.0271; 243.0271
